# The Multidimensional Assessment for Pediatric Patients in Radiotherapy (M.A.P.-RT) Tool for Customized Treatment Preparation: RADAR Project

**DOI:** 10.3389/fonc.2021.621690

**Published:** 2021-03-29

**Authors:** Silvia Chiesa, Elisa Marconi, Nicola Dinapoli, Maria Zoe Sanfilippo, Antonio Ruggiero, Angela Mastronuzzi, Giulia Panza, Annalisa Serra, Mariangela Massaccesi, Antonella Cacchione, Francesco Beghella Bartoli, Daniela Pia Rosaria Chieffo, Maria Antonietta Gambacorta, Vincenzo Valentini, Mario Balducci

**Affiliations:** ^1^UOC di Radioterapia Oncologica, Dipartimento Diagnostica per Immagini, Radioterapia Oncologica ed Ematologia, Fondazione Policlinico Universitario Agostino Gemelli IRCCS, Rome, Italy; ^2^UOS di Psicologia Clinica, Fondazione Policlinico Universitario Agostino Gemelli IRCCS, Rome, Italy; ^3^Università Cattolica del Sacro Cuore, Rome, Italy; ^4^UOC Pediatric Oncology, Department of Woman and Child Health and Public Health, Fondazione Policlinico Universitario A. Gemelli IRCCS, Rome, Italy; ^5^Department of Paediatric Haematology/Oncology, IRCCS Bambino Gesù Children's Hospital, Rome, Italy

**Keywords:** radiotherapy, distress, anesthesia, pediatrics, psychological support, children

## Abstract

**Aims:** Pediatric patients may experience considerable distress during radiotherapy. Combining psychological interventions with standard therapies can reduce the need for sedation. The RADAR Project aims to use a systematic method of recording data that can reveal patients' difficulties and fragility during treatment.

In this context, the aim of our study was to investigate the ability of a multidimensional assessment tool (M.A.P.-RT schedule) to predict the need for sedation during radiotherapy. The schedule, which is administered during the first evaluation, was created to collect information on patients and their families in a standardized way.

**Materials and Methods:** The study enrolled pediatric patients (aged 0–18 years or 18–21 with cognitive impairment). Data were collected by means of the M.A.P.-RT module; this explores various thematic areas, and is completed by the radiation oncologist, psychologist and nurse during their first evaluation. Features were selected by means of the Boruta method (random forest classifier), and the totals of the significant partial scores on each subsection of the module were inserted into a logistic model in order to test for their correlation with the use of anesthesia and with the frequency of psychological support. The results of logistic regression (LR) were used to identify the best predictors. The AUC was used to identify the best threshold for the scores in the evaluation.

**Results:** A total of 99 patients were considered for this analysis. The feature that best predicted both the need for anesthesia and the frequency of psychological support was the total score (TS), the AUC of the ROC being 0.9875 for anesthesia and 0.8866 for psychological support.

**Conclusion:** During the first evaluation, the M.A.P.-RT form can predict the need for anesthesia in pediatric patients, and is a potential tool for personalizing therapeutic and management procedures.

## Introduction/Background

From 2001 to 2010, the worldwide incidence of cancer in subjects aged 0–19 years was 155.8 per million/year (1). In Italy, the AITRUM has estimated that 5-year survival improved among 11,000 children and adolescents newly diagnosed between 2016 and 2020 (2).

A current aim of medicine is to offer therapeutic responses in accordance with the international recommendations, while at the same time enrolling young patients in centralized clinical research protocols in order to guarantee homogeneous, high-quality treatment.

Radiotherapy (RT) is one of the therapeutic options for pediatric neoplasms. In order to ensure the accuracy of radiation treatment, it is necessary to create an *ad-hoc* immobilization system that is personalized according to the site of the tumor. Pediatric patients are required to cooperate closely, first during treatment preparation (by remaining motionless during the preparation of the immobilization system and the phase of image acquisition) and then in the phase of treatment delivery.

Therefore, RT is not only a challenge for children but also for parents and healthcare professionals (3–7). When patients are unable to maintain a fixed and reproducible position, sedation or general anesthesia (GA) becomes necessary. This means that children and adolescents undergo numerous changes in their lifestyle, daily activities, and school and social activities (8). Moreover, numerous sedation treatments can have an impact on eating habits, in that patients have to fast for several hours. Finally, repeated sedation, high doses of sedatives, the use of multiple drugs and general anesthesia can increase the risk of medical complications (9).

In the literature, several studies have described the benefit of combining psychological support interventions with standard therapies for reducing the number of sedations (6, 9, 10). In pediatric RT, recent research has revealed the importance of both pharmacological and non-pharmacological interventions. However, the approaches adopted vary markedly and few assessment tools are available for doctors and researchers. For example, there is, as yet, no assessment tool that can quickly select the type of intervention needed.

In this regard, it has been ascertained that a multi-disciplinary approach implemented by a specialized team (4, 11) can identify the individual patient's needs and enable targeted interventions to be undertaken in order to facilitate treatment preparation and improve patient compliance, thereby avoiding sedation whenever possible.

The RADAR project aims to increase the level of personalization of radiotherapy for the pediatric patient through a multidimensional approach. The project utilizes a standardized tool (Multidimensional Assessment for Pediatric Patients in Radiotherapy M.A.P.-RT schedule) to collect information on patients and their families during the first clinical evaluation; the results obtained allow clinicians to predict the need for sedation and the intensity of specific psychological preparation, and to plan supportive treatment.

## Materials and Methods

### Design, Setting, and Inclusion Criteria

This pilot observational study enrolled pediatric patients with an oncological diagnosis for which radiation treatment had been prescribed. During the patient's first examination, the radiation oncologist, the nurse and the psychologist filled in the M.A.P.-RT form (Table 1), a multidimensional assessment form covering a selection of items and standardized tests.

**Table 1:** M.A.P.-RT scoring and items details.

	**Dimension**	**Contents**	**Score**
**A**	**Pain/distress**	**Nursing observation**	**0–10**
**B**	**Age**	**Discomfort/age scoring**	**0–10**
**C**	**Medical**	**First medical evaluation**	**0–22**
		C1: Family information on diagnosis	0–2
		C2: Information on the purpose of RT	0–2
		C3: Information shared with the patient	0–2
		C4: Collaboration of the patient with previous radiography	0–2
		C5: Collaboration with requests from parents/health workers	0–2
		C6: Collaboration in separation from parents	0–2
		C7: Distress/pain level detected during the visit	0–10
**D**	**Physical**	**Report on skills for RT**	**0–26**
		D1: Physical difficulties	0–2
		D2: Cognitive difficulties	0–2
		D3: Language difficulties	0–2
		D4: Minutes required for the RT	5–20
**E**	**Emotional distress**	**First Entry to Linac Room (CEMS Scale: Children's Emotional Manifestation Scale)**	**0–25**
		E1: Facial expression	1–5
		E2: Vocalization	1–5
		E3: Activity	1–5
		E4: Interaction	1–5
		E5: Level of cooperation	1–5
**F**	**Psychological**	**Psychological interview**	**0–30**
		F1: Psychological difficulties before diagnosis	0–3
		F2: Recent loss of mobility/autonomy	0–3
		F3: Patient distress reactive to diagnosis	0–3
		F4: Patient externalizing problem	0–3
		F5: Patient internalizing problem	0–3
		F6: Patient's fear/anxiety (last 2 weeks)	0–3
		F7: Parent's fear/anxiety (last 2 weeks)	0–3
		F8: Parenting difficulties	0–3
		F9: Family/Social/Work difficulties	0–3
		F10: Traumatic events before diagnosis	0–3
		**Total Score M.A.P.-RT**	**0–123**

*The values without bold are the ranges of the scores of the single items. The values in bold are the ranges of the total scores of the contents A–F*.

A preliminary set of data were collected from patients who accessed our center last year.

The form was administered to two groups of patients: one aged between 0 and 18 years and the other between 18 and 21 years with cognitive impairment. The M.A.P.-RT form provides for the calculation of several partial scores and of a final score, which could be useful in order to understand the various patients' RT-related needs. However, in this phase, the patient's care pathway did not undergo changes following the scoring of the instrument; patients underwent the preparation phase of the treatment with or without specific interventions and sedation, according to their needs.

#### Multidimensional Assessment for Pediatric Patients in Radiotherapy, M.A.P.-RT Schedule

The crucial areas in the preparation of children for radiotherapy were identified after 2 years of clinical observations by RT care providers and a psychologist, and on the basis of the literature.

**Figure 1:**
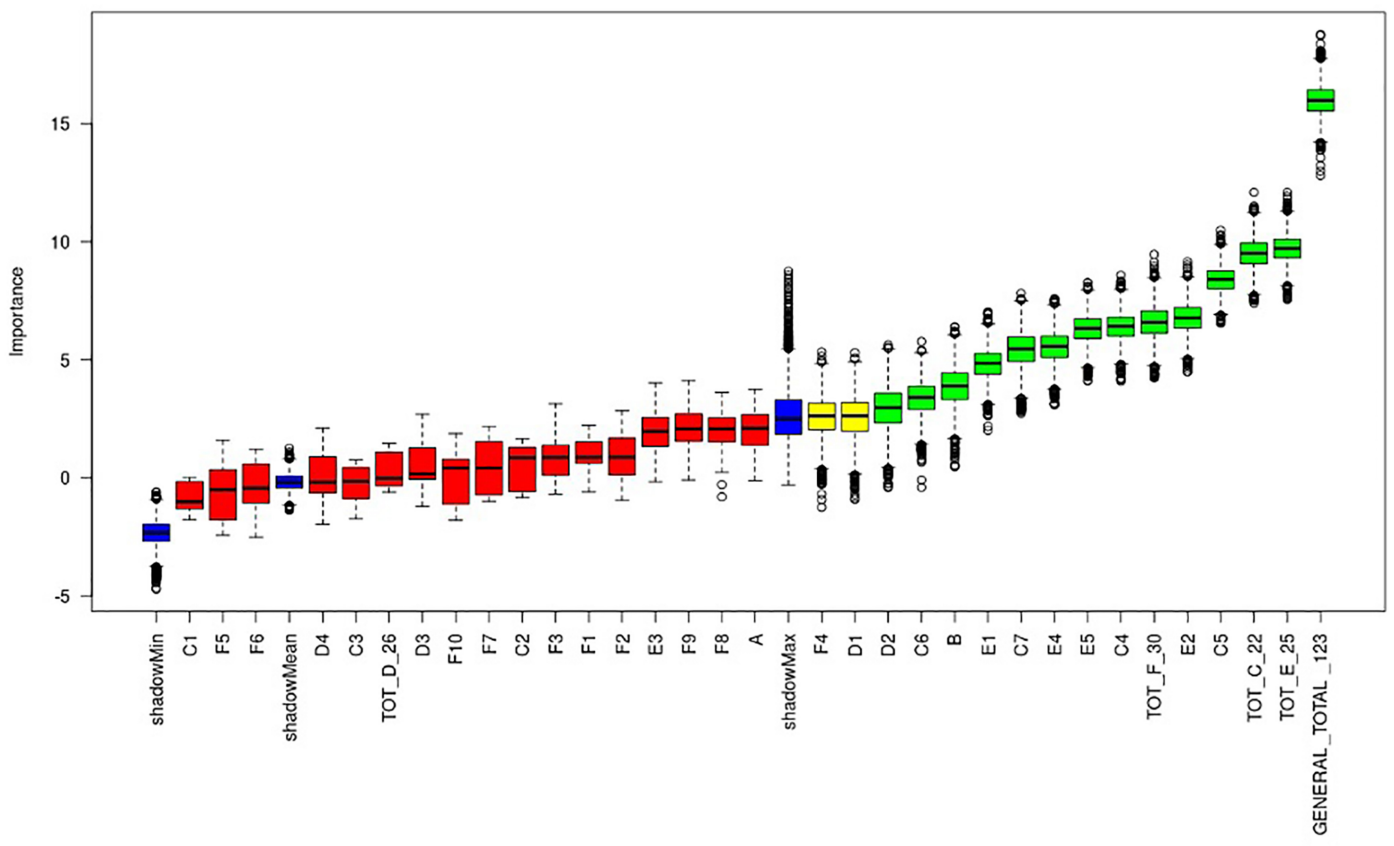
Plot of Boruta feature selection process for the “anesthesia” outcome: the red boxes represent the not relevant items, the yellow are the uncertain ones, the green are the relevant items. Blue boxes are calculated as reference levels during the run of Boruta algorithm.

Six areas of interest were identified (Table 1 and Figure 1):

- **Pain distress-Nursing observations:** data collected when the patient enters the Unit for RT examination for the first time. These data are collected by the first nurse who meets and welcomes the child (Wong Baker Scale WBS—observer: 0–10); maximum value: 10;
- **Development-Discomfort age score:** this score indicates the potential impact of age on the overall score in children from 0 to 10 years, due to the characteristics of the development and growth of children; maximum value: 10;
- **Medical-First Medical Evaluation:** this area collects data on the amount of information provided and on the degree of collaboration and distress/pain noted by the RT specialist while taking the medical history and performing clinical evaluation; maximum value: 22;
- **Physical-Skills for RT:** these are the specific skills required for the planned RT treatment, with particular regard to positioning and maintenance of posture during the RT fraction; maximum value: 26;
- **Emotional Distress-First entry to LINAC room (CEMS):** behavioral and emotional reactions of the child on first entering the bunker; technician records by means of the Children's Emotional Manifestation Scale—CEMS; maximum value: 25;
- **Psychological-Psychological interview:** this area reports scores assigned by the psychologist following the assessment of psychological areas that are deemed to influence the patient's compliance with RT in both the preparation and treatment phases; for example, past or current mental disorders, previous traumatic events, patient's tendency to implement internalizing or externalizing strategies in the management of distress/pain, family difficulties, etc. maximum value: 30.

### Statistical Analysis

We defined two outcomes: anesthesia and psychological support. *Anesthesia* was defined as a binary outcome (patients who needed anesthesia and patients who did not); *psychological support* was defined as a binary outcome (patients who needed an *intensive* course of psychological support and patients who did not); psychological support was defined as “intensive” or “standard” according to a threshold value of 1 support session every 3 days, which was calculated by dividing the median number of psychological sessions during the period of treatment by the number of RT fractions.

Modeling process was assessed in two phases: (12) we performed the feature selection among all the items of M.A.P. report using the Boruta method, which is a novel feature-selection algorithm for identifying all significant variables, designed as a wrapper around a random forest classification algorithm. This test iteratively removes those features which prove to be less significant than random probes, thereby yielding a series of graphs that contain a *z*-score. The *p*-value of the *z*-test is 0.01. All features (items in the M.A.P. report) were tested against the two outcomes listed above (13). The model chosen was the Elastic Net, a regularized generalized linear model; it was used because it enables to discriminate among the items more accurately and to pick out the most significant covariates, even in the presence of cross-correlation. With regard to the outcome “*anesthesia*,” our statistical model showed the population classified by a score related to the most significant items found in the M.A.P.-RT schedule: negative scores, i.e., lower than 0, were related to “no anesthesia,” while positive ones were found to be mostly associated to “yes anesthesia.” The value of each item, when multiplied by the coefficients and added together (linear predictor), returns a score that indicates the actual correlation between patients' characteristics and their need for anesthesia. The same process was used to identify patients who needed *intensive* psychological support and those who did not. The validation of the models has been performed by using area under the receiver operating characteristic curve (AUC) and confusion matrix statistics computing accuracy, McNemar's test *p*-value, 95% C.I. *p*-value, *k* statistic and *P*-value of binomial test to see if the accuracy is better than “no information rate” (accuracy > NIR).

### Ethics Approval

This study was approved by the ethics committee of Agostino Gemelli Polyclinic, Rome.

## Results

The study involved 99 consecutive pediatric cancer patients (M.51; F:48), who underwent 99 RT courses (n° RT Fractions: 2097) between January and December 2019.

The M.A.P.-RT form was administered to all patients and data were collected for each area; 20 children who underwent retreatment were excluded from the study. Twenty-two patients needed sedation during radiation treatment. One patient (age 21) presented cognitive impairment. The median age was 7.5 years (range 1–21). Patients' characteristics are reported in Table 2.

**Table 2:** Descriptive statistics and epidemiology of patients population.

	**Total**	**%**
**Total number patients**	99	100%
**Sex**		
Male	51	51.5%
Female	48	48.5%
**Histological diagnosis**		
Brain neoplasm	42	42.4%
Hematological neoplasm	24	24.2%
Sarcomas	14	14.1%
Wilms tumor	2	2.0%
Nephroblastoma	2	2.0%
Neuroblastoma	15	15.2%
**RT site**		
Brain	45	45.5%
Abdomen	15	15.2%
Thorax	9	9.1%
Pelvis	6	6.1%
ACS (Spinal-Skull-Axis)	8	8.1%
TBI (Total Body Irradiation)	11	11.1%
Other	5	5.1%
**Immobilization system**		
Thermoplastic mask	53	53.5%
VAC-LOC (Vacuum Locked)	21	21.2%
Wing board	9	9.1%
Other	16	16.2%

The team involved in data collection was made up of two oncological radiotherapists, a child psychologist, a nurse, a technician and a resident.

Fourteen items proved to be predictive of the need for anesthesia: B, C4, C5, C6, C7, TOT C, D2, E1, E2, E4, E5, TOT E, TOT F, GENERAL TOT (Figure 1). The general total score, and, in particular, the total score of the first clinical medical evaluation and of emotional distress seemed to be the most predictive dimensions of the schedule. The single items that proved predictive were: patient age (see point B Table 1), level of collaboration of the patient during previous diagnostic tests (see point C4 Table 1), level of the patient's collaboration with the requests of parents and health workers (see point C5 Table 1), level of collaboration during separation from the parent (see point C6 Table 1), level of distress/pain detected during the first visit (see point C7 Table 1), and cognitive difficulties and/or deficits (see point D2 Table 1). Other variables that proved to be highly predictive were those related to the patient's psycho-physical attitudes at the time of first entry to the therapy room, specifically: the patient's facial expression (see point E1 Table 1), vocal expressions such as crying and screaming (see point E2 Table 1), the patient's interaction through verbal or non-verbal responses or possible absence of interaction (see point E4 Table 1), the patient's level of cooperation, i.e., whether the child actively participates or is indifferent to external requests (see point E5 Table 1).

**Figure 2:**
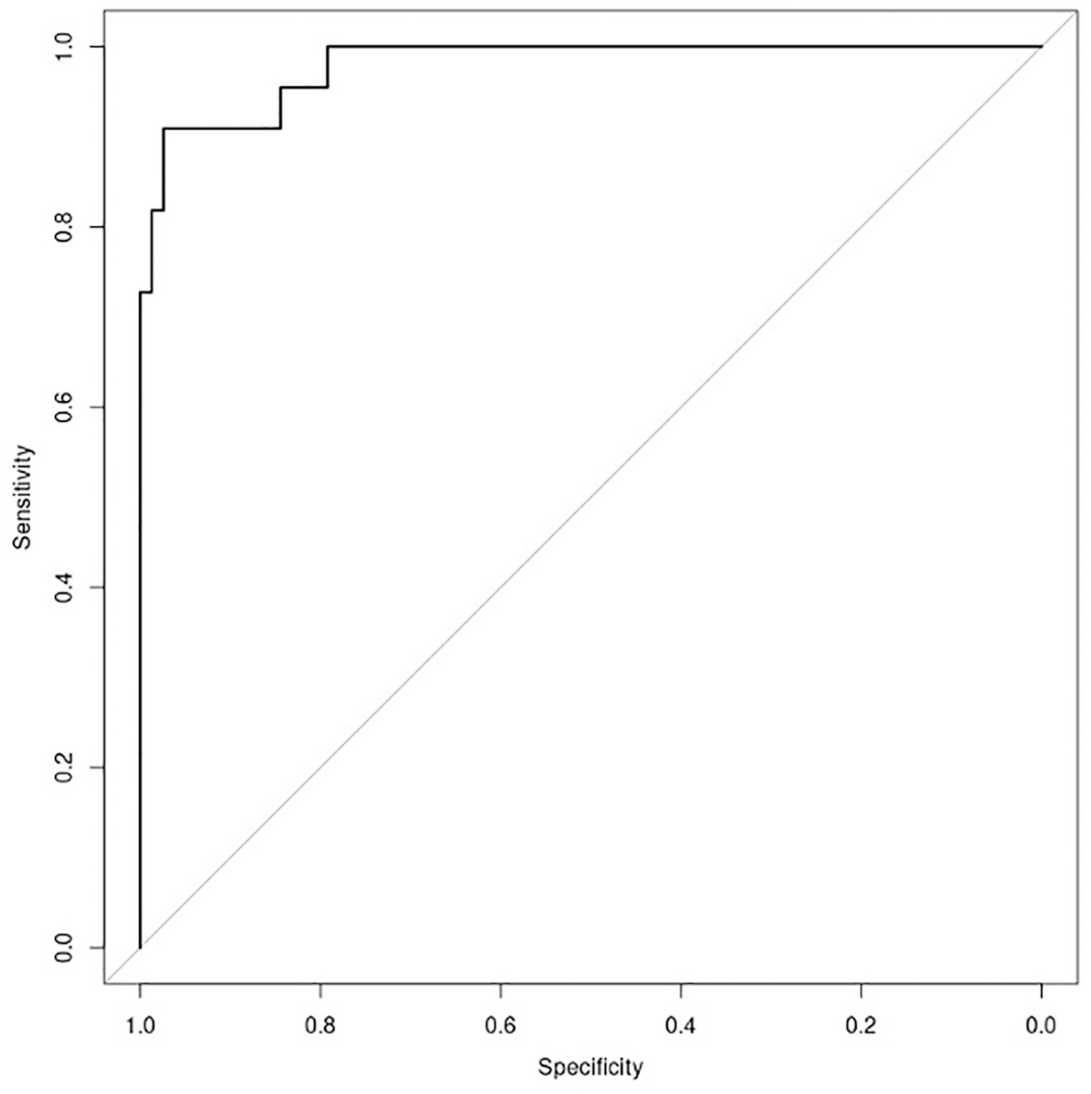
Plot of ROC curve for the “anesthesia” model (AUC = 0.9875), showing possibility to identify patients who need anesthesia support with regards to the total score, achieved putting into the model the value of relevant items of M.A.P.-RT.

**Figure 3:**
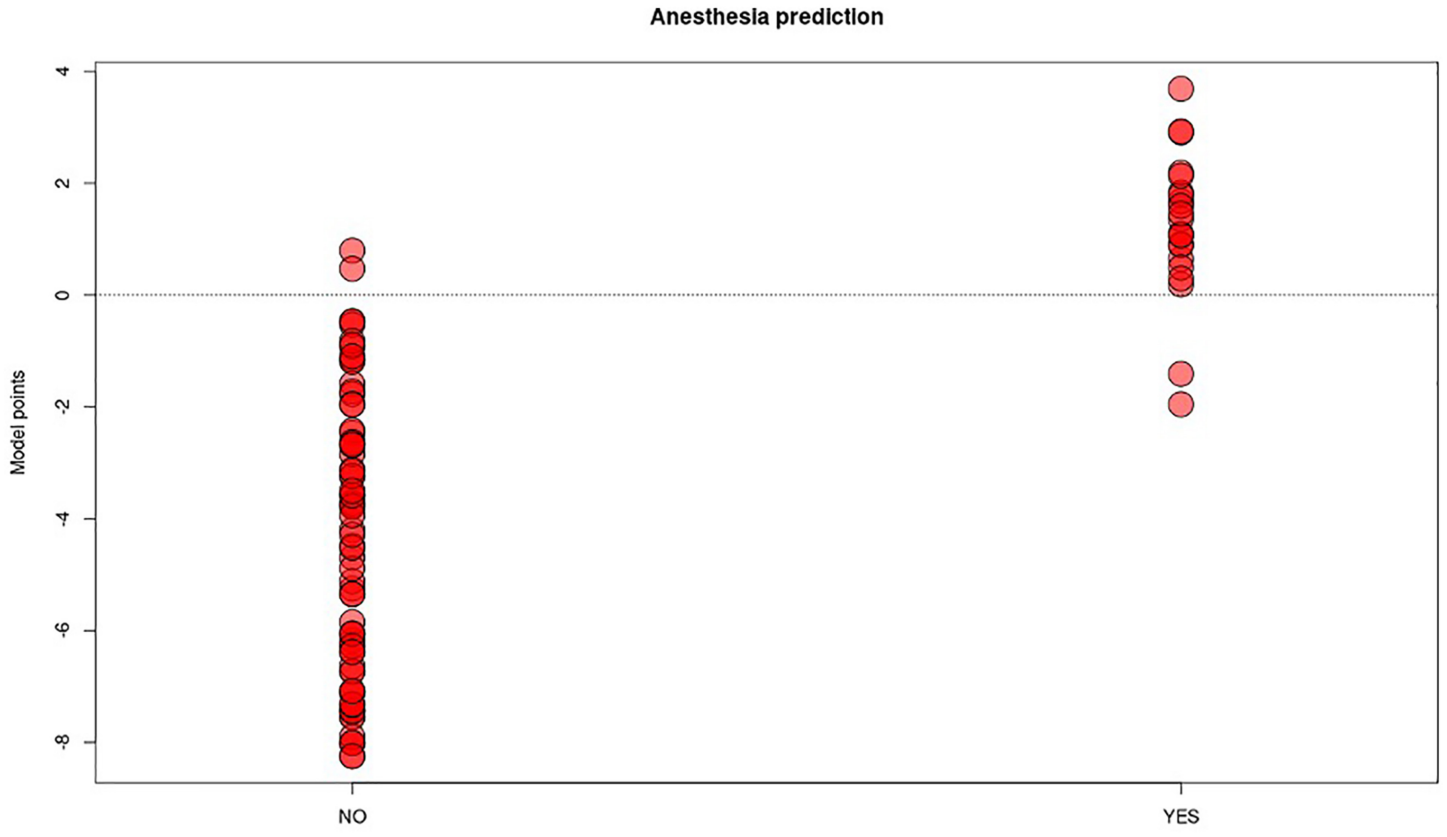
Distribution plot of “anesthesia” predictive model score (y axis) in the two groups of patients undergoing (YES) or not (NO) to anesthesia procedure. The red dots represent each patient in the two groups, the threshold line chosen to best split the two categories is the score 0.

With regard to the TS, the AUC of the ROC was 0.9875 (sensitivity = 0.91, specificity = 0.97) with a *p*-value = 7.986−12 (Figures 2, 3). Negative results in Figure 3 identified patients who underwent treatment without sedation, and positive results identified patients undergoing anesthesia. Confusion matrix statistics are summarized in Tables 3, 4.

**Table 3:** Model “anesthesia” performance table: the model shows very high accuracy (0.96), sensitivity (0.91), and specificity (0.97).

**Confusion matrix and statistics for anesthesia prediction model**			
	**Outcome** **+**	**Outcome -**	**Total**
Test +	20	2	22
Test -	2	75	7
Total	22	77	99
No information rate: 0.78			
*P*-value [Acc > NIR]: 4.567e-07			
Kappa: 0.88			
Mcnemar's Test *P*-value: 1			
**Point estimates and 95 % Cis**			
Accuracy	0.96 (0.90, 0.99)		
Apparent prevalence	0.22 (0.14, 0.32)		
True prevalence	0.22 (0.14, 0.32)		
Sensitivity	0.91 (0.71, 0.99)		
Specificity	0.97 (0.91, 1.00)		
Positive predictive value	0.91 (0.71, 0.99)		
Negative predictive value	0.97 (0.91, 1.00)		
Positive likelihood ratio	35.00 (8.86, 138.31)		
Negative likelihood ratio	0.09 (0.02, 0.35)		

**Table 4:** Model “psychological support” performance table: the model shows fair accuracy (0.88), optimal sensitivity (0.91), and specificity (0.94). Overall performance is slightly lower than the “anesthesia” model.

**Confusion matrix and statistics for psychological support prediction model**			
	**Outcome** **+**	**Outcome –**	**Total**
Test +	50	7	57
Test -	5	37	42
Total	55	44	99
No information rate: 0.56			
*P*-value [Acc > NIR]: 4.078e-12			
Kappa: 0.75			
Mcnemar's test *P*-value: 1			
**Point estimates and 95 % Cis**			
Accuracy	0.88 (0.80, 0.94)		
Apparent prevalence	0.58 (0.47, 0.67)		
True prevalence	0.56 (0.45, 0.66)		
Sensitivity	0.91 (0.80, 0.97)		
Specificity	0.84 (0.70, 0.93)		
Positive predictive value	0.88 (0.76, 0.95)		
Negative predictive value	0.88 (0.74, 0.96)		
Positive likelihood ratio	5.71 (2.88, 11.33)		
Negative likelihood ratio	0.11 (0.05, 0.25)		

**Figure 4:**
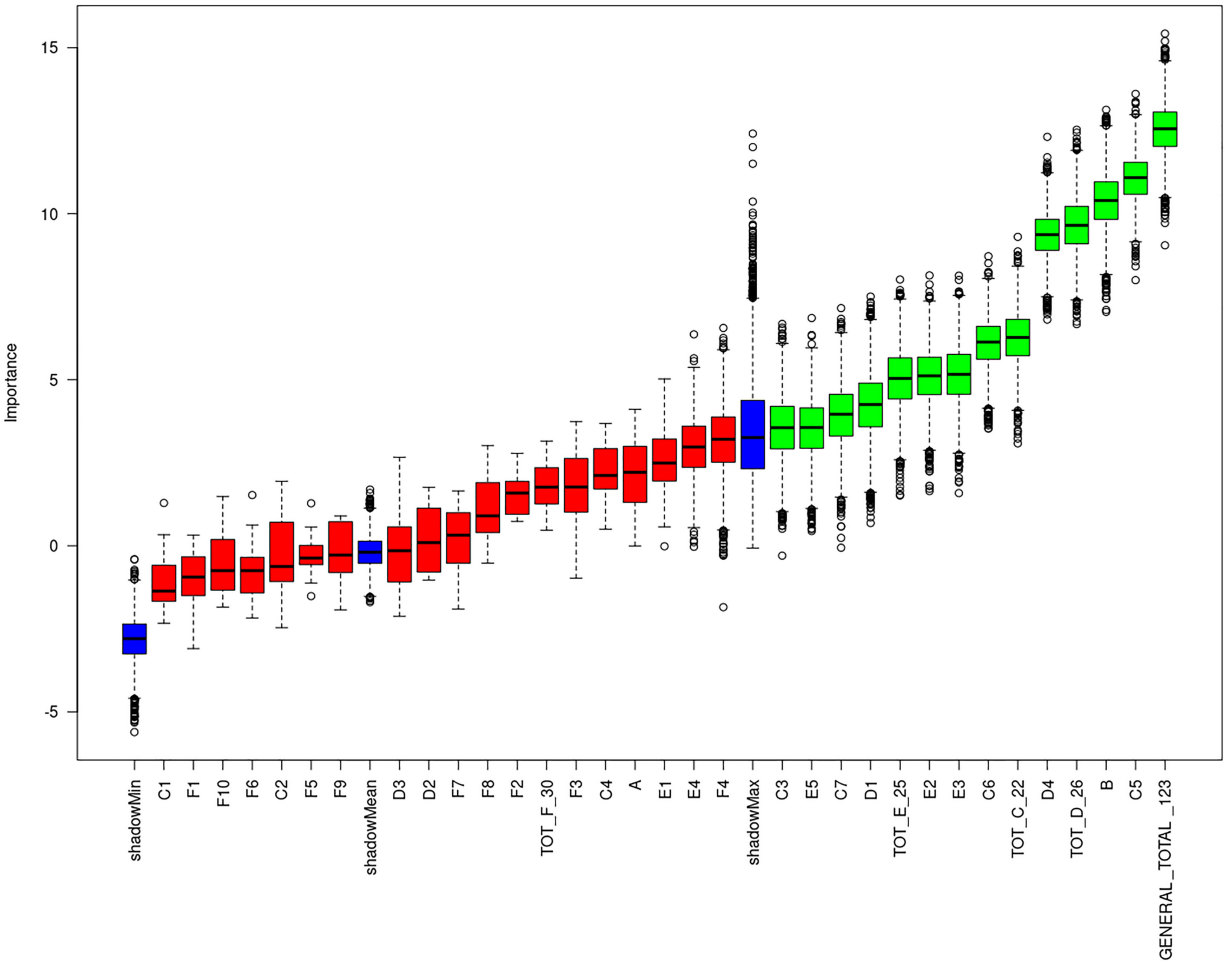
Plot of Boruta feature selection process for the “intensive psychological support” outcome: the red boxes represent the not relevant items, the yellow are the uncertain ones, the green are the relevant items. Blue boxes are calculated as reference levels during the run of Boruta algorithm.

We also analyzed the intensity and frequency of psychological support provided for patients. During 99 RT Courses, corresponding to 2097 fractions, 766 psycho-educational interventions were implemented (median: 6, range: 1–20). Moreover, 46 patients received intensive psychological support (>1/3) and 53 standard support (<1/3). In this secondary analysis, the items displaying a predictive value regarding the intensity of the psychological support to be provided were: B, C3, C5, C6, C7, TOT C, D1, D4, TOT D, E2, E3, E5, TOT E, GENERAL TOT (Figure 4). The information obtained from points C4–C7, which concern the patient's collaboration, detachment from the caregiver and level of distress/pain, seemed to correlate more closely than the others (C1–C3) with the need for psychological support. Age was also confirmed as a fundamental parameter.

**Figure 5:**
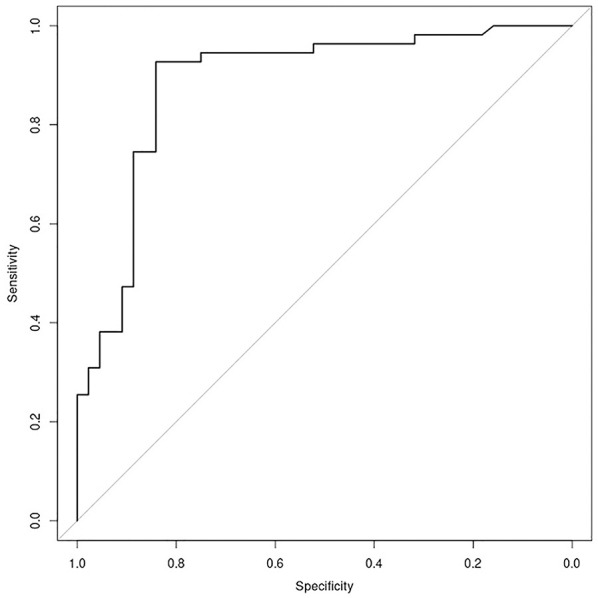
Plot of ROC curve for the “psychological support” model (AUC = 0.8866), showing possibility to identify patients who need intensive support with regards to the total score, achieved putting into the model the value of relevant items of M.A.P.-RT.

**Figure 6:**
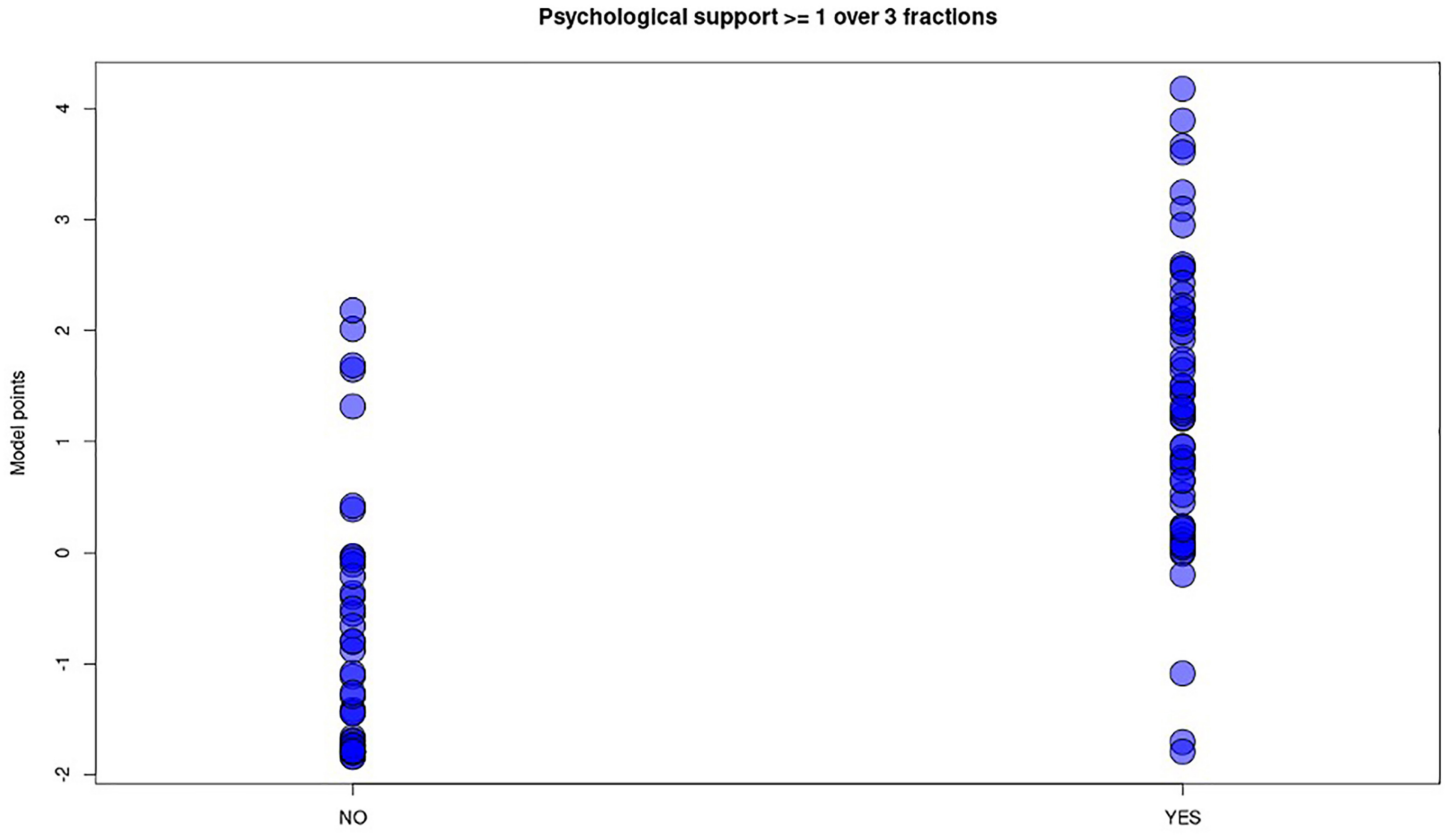
Distribution plot of “psychological support” predictive model score (y axis) in the two groups of patients undergoing (YES) or not (NO) to intensive psychological support. The blue dots represent each patient in the two groups. Differently from “anesthesia” model no threshold line has been plotted, being wide overlapping between the two categories of patients despite fair model performances.

In this case, the model was less accurate; the AUC of the ROC was 0.8866, (sensitivity = 0.91, specificity = 0.84) with a *p*-value = 2.122−07, therefore indicating a higher risk of false negatives or false positives (Figures 5, 6).

## Discussion

In pediatric oncology, RT, alone or in combination with surgery or chemotherapy, has become an important treatment option for several kinds of neoplasms. Although it does not cause any pain, the young patient must remain alone and motionless for several minutes during the time of irradiation, a situation that frequently causes distress reactions. Distress reactions are also aggravated by previous traumatic events experienced by patients and their families, such as painful experiences in hospital or at the hands of other healthcare personnel. Such conditions can make the new treatment even more traumatic for the child. Changes in the child's routine, owing to the need to attend the hospital daily, can also constitute an additional source of distress, which may determine the need for sedation. In the literature, some studies have described the experience of anxiety and distress of patients and parents in Pediatric Hematology/Oncology Units, while few studies have described the situation in RT units (7).

In children undergoing radiotherapy, anesthesia ensures adequate immobilization during verification of the patient's position and therapy delivery. With regard to acute and late risks, daily treatment under sedation (14) is associated with major changes in the child's daily routine, such as the need to fast for several hours (15).

Carrying out radiation treatment under sedation also involves specific and careful organization of the treatment room and the presence of the anesthesiologist and the nurse for the entire duration of the treatment; this increases the occupation time of the room and inevitably raises healthcare costs (9).

Some studies in the literature have shown the benefit of combining psychological interventions with standard therapies (11), while others have analyzed the specific activities that can be planned in order to help children to cooperate with RT (16); indeed, personalizing the child's preparation can reduce the need for sedation during radiation treatment (10). This problem has often been addressed in the literature, which has highlighted the need for specific scales that can record the levels of pain, anxiety and anguish of pediatric patients and their approach to radiation treatment under sedation. Several instruments have been used to predict pediatric distress in radiotherapy: the Behavioral Distress Observation Scale (OSBD) (8), parent report (7), and qualitative interview (17); other instruments have been applied more generally in the context of sedation (18) or during invasive medical procedures (19).

These tools have made it possible to carry out interventions to reduce emotional distress or monitor the sedation of the pediatric patient. Specifically, some studies have shown that adequate preparation through play activities and the presence of a child psychologist are effective in reducing anxiety and negative emotions (10, 20). For example, some authors have reported that engaging the patient in play activities at the hospital reduces negative emotions and lowers anxiety levels in comparison with normal care (21, 22). Moreover, psychological interventions have proved effective in reducing RT-related distress (as measured by heart rate) and have also been seen to be useful specifically in pediatric radiotherapy (23).

However, few studies have provided clinicians with effective tools to guide the therapeutic and care pathway of pediatric cancer patients undergoing RT. Inspired by the universally used bio-psychosocial model proposed by A. E. Kazak (24), the RADAR project aims to monitor and collect information on pediatric patients undergoing RT and to provide a rapid assessment tool that can identify the needs and risks of these patients.

In our center, the child psychologist, when required, plans psycho-educational intervention before the simulation of radiation treatment. The support plan includes weekly psychological sessions during the period of treatment. Patients defined as more complex, or who have crises during treatment, undergo several interventions per week, daily if necessary.

The introduction of the M.A.P.-RT schedule facilitates multidisciplinary assessment in the initial stages of treatment. To our knowledge, this is the first schedule to collect data in a standardized way in pediatric RT, in order to identify patients' needs; it can therefore optimize and personalize psychological support.

The results of our study identified those items that were predictive of the need for sedation during radiotherapy and for intensive psychological support. Some items were found to be common to both analyses, and proved to be fundamental parameters. For example, age (point B Table 1) was confirmed as a fundamental parameter; in our study, we did not establish an age cut-off, but younger children are usually those who most need to be sedated during treatment. In the literature, the study by Linda Scott, Fiona Langton and Joan O'Donoghue (25) considered 63 children aged between 2 and 5 years; their outcome data suggested that sedation could be minimized in this age-group through the implementation of an effective play preparation program.

Other predictive items were found in group C (“first medical evaluation”): the patient's degree of cooperation, the degree of cooperation in separation from parents, and the distress/pain level detected during the visit. In addition, the total score of F (“psychological interview” and the items in group E, the CEMS scale, had a strong impact on our schedule. The CEMS has been validated (26) and is already used in the field of pediatric radiotherapy (27). Recording patients' distress on entering the bunker for the first time helps the clinician and psychologist to immediately ascertain the complexity of the individual patient.

Patients who have initially cooperated may experience new side-effects, such as fatigue or crises, during treatment, and may therefore need subsequent psychological support and increased occupancy machine time. By contrast, patients who have difficulty in the preparation phases may rapidly overcome their initial fears or difficulties and require fewer interventions by the child psychologist.

To our knowledge, the M.A.P.-RT schedule is the first tool that has proved to be accurate in predicting, during the first clinical evaluation, the need for sedation during radiation treatment. In addition, it is well-known that administering RT to children requires a great deal of cooperation and that a specialized multidisciplinary team is necessary; the M.A.P.-RT schedule could allow quick, simple and codified communication among the members of this team.

Indeed, the use of the M.A.P.-RT schedule would enable the radiotherapist, child psychologist, residents, nurse and RT technician to understand the specific needs of the patient and to tailor their interventions during treatment preparation and delivery.

Another achievement of this first phase of modeling is that we were able to discern which items in the M.A.P.-RT schedule were the most important. Indeed, some items were not effective in predicting the need for anesthesia during treatment. Therefore, in the future, the form could be modified in such a way as to include only those items with the greatest predictive capability. This would reduce the time needed for its compilation, making it more manageable by the various professionals involved. Of course, the shorter version would need to be validated on a larger sample of patients, such as the sample that we are currently enrolling prospectively.

A limitation of our study is the fact that the tool does not accurately predict the intensity of the psychological support to be provided; this is partly because, during RT, the need for psychological support may change over time, owing to sideeffects, the influence of other sources of distress, interactions with drugs, clinical or family difficulties, etc. For this reason, psychological support during RT must be modulated over time and personalized, in terms of both type and frequency, according to the patients needs.

In the light of these observations, other scales or tools could be used to evaluate the evolution of patients' needs over time. In this regard, the Radar Project envisions the use of other tools that allow further evaluations during RT (daily evaluation by RT technicians; medical evaluation during RT, psychological scales, etc.). We think that, if the M.A.P.-RT schedule were integrated with data subsequently collected during RT, it would be possible to further customize psychological support, adjust it over time and verify its effectiveness.

## Conclusions

The study shows that multidimensional assessment is an optimal strategy during the procedures of radiation oncology setup and RT delivery in pediatric patients. In particular, the M.A.P.-RT schedule proved to be a good and appropriate work tool capable of predicting, even at the time of the first visit, the pediatric patient's need for sedation. Moreover, its use could also improve cooperation among the specialists of the pediatric team.

## Data Availability Statement

The raw data supporting the conclusions of this article will be made available by the authors, without undue reservation.

## Ethics Statement

The studies involving human participants were reviewed and approved by Ethics Committee - Fondazione Policlinico Universitario Agostino Gemelli, IRCCS. Written informed consent to participate in this study was provided by the participants' legal guardian/next of kin.

## Author Contributions

MB, SC, EM, and MS conceived of the presented idea. ND processed the experimental data, performed the analysis, and designed the figures. MS collected data and drafted the manuscript, while SC and EM reviewed the text. MB provided final approval of the version to publish. All authors discussed the results and commented on the manuscript.

## Conflict of Interest

The authors declare that the research was conducted in the absence of any commercial or financial relationships that could be construed as a potential conflict of interest.
